# Obstructive sleep apnoea and anaesthesia

**DOI:** 10.4103/0972-5229.43680

**Published:** 2008

**Authors:** A. Rudra, S. Chatterjee, T. Das, S. Sengupta, G. Maitra, P. Kumar

**Affiliations:** **From:** Apollo Gleneagles Hospital, Kolkata, India; 1 Apollo Medical College and Hospital, Kolkata, India

**Keywords:** Anaesthesia, complications, obstructive sleep apnoea, perioperative care

## Abstract

Obstructive sleep apnoea (OSA) correlates positively with obesity and age, both of which are becoming increasingly prevalent. Obstructive sleep apnoea occurs much more frequently in clinical practice than formerly diagnosed, and that this condition represents complex challenges for difficulty in mask ventilation, laryngoscopic intubation, accelerated arterial desaturation, postoperative monitoring and discharge status. In this review article pathophysiology, diagnosis, and perioperative management of this group of patients have been discussed in detail.

Obstructive sleep apnoea (OSA) is an increasingly common sleep disorder, which is of particular concern to anaesthesiologists because it is associated with increased perioperative morbidity and mortality. Because OSA is undiagnosed in an estimated 80 percent of patients, it is necessary that anesthesia practitioners have adequate knowledge of the clinical presentation and diagnosis of OSA.[1] With increasing number of surgical procedures performed on an outpatient basis, there are an increasing number of patients with OSA scheduled for ambulatory surgery. The suitability of ambulatory surgery in OSA patients remains controversial however.

The concerns in patients with OSA include potent upper-airway obstruction, difficult tracheal intubation and postoperative respiratory depression and airway obstruction. The scientific literature regarding the perioperative management of OSA patients is sparse and of limited quality. Therefore, the purpose of this review article is to improve the perioperative care and reduce the risk of adverse outcomes in patients with OSA who receive sedation, analgesia or anesthesia for diagnostic or therapeutic procedures under the care of an anaesthesiologist or intensivist.

## Definition

Obstructive sleep apnoea is a syndrome characterized by periodic, partial or complete obstruction of the upper airway during sleep.[2]

## Prevalence

Obstructive sleep apnoea has been recognized as a major contributor to morbidity and mortality in developed countries. However, its impact in developing countries is only now being appreciated.

In India, Udwadia and colleagues[3] studied urban men between 35-65 years of age presenting to the hospital for routine check up, and reported the estimated prevalence of OSA as 19.5 percent. Sharma and colleagues[4] recently reported the prevalence of OSA in male is 19.7 percent and in female is 7.4 percent.

In USA, obstructive sleep apnoea is common in the general population and its incidence has been estimated between two to five percent of middle-aged adults, and perhaps more than 10 percent of those older than 65 years of age.[5–7] OSA affects a similar proportion of the population as asthma or diabetes, which makes it a significant public health problem.[8,9] Some subgroups have a greatly increased incidence of OSA. For example, it is estimated that commercial truck drivers have a 46 percent incidence of OSA and professional football players a 14 percent incidence of OSA.[10,11] Anecdotal reports of adverse respiratory events in patients with OSA raise the concern that it is a factor that increases perioperative risk.[12]

## Pathophysiology (in brief)

Sleep apnoea occurs when the negative airway pressure that develops during inspiration is greater than the muscular distending pressure, thereby causing airway collapse. Obstruction can occur throughout the upper airway; above, below, or at the level of uvula.[13,14] Because there is an inverse relationship between obesity and pharyngeal area, the smaller size of the upper airway and pharyngeal area in the obese patient causes a more negative pressure to develop for the same inspiratory flow.[15,16] Kuna and Sant'Ambrogio[15] have also postulated that there may be a neurogenic basis for the disease in that the neural drive to the airway dilator muscles is insufficient or not coordinated appropriately with the drive to the diaphragm. Obstruction can occur during any sleep state but is often noted during rapid eye movement (REM) sleep. Nasal continuous positive airway pressure (CPAP) can ameliorate the situation by keeping the pressure in the upper airways positive, thus acting as a ‘splint’ to maintain airway patency.

**Figure 1:**
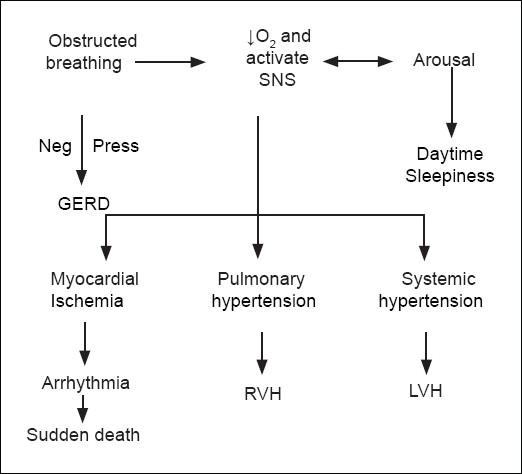
SNS: Sympathetic nervous system: RVH & LVH: Right and Left ventricular hypertrophy, Neg.Press: Negative pressure, GERD: Gastro esophageal reflux disease

If the loss of pharyngeal muscle tone and pharyngeal collapse are partial, but still great enough to cause the inspired air to flutter around the uvula and / or the tongue and / or the epiglottis, there will be *snoring* and *hypopnea.*[2] The presence of apnoea and hypopnea during sleep is called sleep-disordered breathing (SDB). In order to survive each obstructive episode, the patient has to have some sort of arousal (*mini-arousal*) due to increased inspiratory effort and the response to arterial hypoxemia and hypercarbia.[17] Each arousal causes sympathetic nervous system stimulation, which, in turn causes sympathetic and pulmonary hypertension as well as myocardial ischaemia [Figure 1].[13] The increased intrathoracic pressure can cause reflux from the positive-pressure stomach to the negative pressure esophagus.

### Causes Anatomical

Central (neck) obesity
Micro-or retrognathia
Maxillary underdevelopment (Treacher-Collins Syndrome)
Pharyngeal encroachment (tonsillar hypertrophy, large tongue, acromegaly, tumors, edema)

### Neuromuscular

Bulbar palsies
Neurological degenerative disorders (multi-system atrophy)
Myopathies (Duchenne dystrophy)

### Other provoking factors

Alcohol
Sedative drugs
Sleep deprivation
Increased nasal resistance
Hypothyroidism

If a cause of OSA is present, it is reasonable to investigate whether OSA exists.

## Symptoms and Signs

Symptoms of OSA derive at least from the consequences of obstructed breathing during sleep. The individual experiences cycles of sleep, obstruction, arousal, restoration of breathing and falling asleep again. This results in poor quality sleep. Patients with OSA commonly report increased daytime sleepiness. Furthermore, individuals with OSA may have morning headaches.[18]

Clinical diagnosis of OSA begins with an awareness of how common it is and consideration of its presence even in young and otherwise healthy individuals.[19] The clinical sensitivity of the diagnosis is only about 60 percent at best and the confirmation of the disease requires laboratory and other special testing.[20] Women typically have fewer signs and symptoms of OSA than men, although they may complain more of fatigue, tiredness, or lack of energy rather than sleepiness.[21–23] The constellation of snoring, excessive daytime somnolence, a body-mass index more than 35, and observed apnoea during sleep strongly suggest the diagnosis. Adding to the likelihood of the diagnosis are observations by a sleep partner of overt choking or gasping episodes (i.e. actual obstruction) in addition to apnoea, a neck circumference > 17 inches and associated hypertension.[24] Features of OSA differ in the adult and children [Table 1].[25,26]

**Table 1:** Differences between adult and children OSA

Features	Adult	Child
Snoring	Intermittent	Continuous
Mouth breathing	Uncommon	Common
Obesity	Common	Uncommon
Daytime hypersomnolence	Common	Uncommon
Gender prediction	Male	None
Most common obstructive event	Apnoea	Hypopnea
Arousal	common	Uncommon
Treatment		
Non surgical	CPAP in majority	CPAP in minority
Surgery	Selected cases	T and A in majority

CPAP: Continuous positive airway pressure, T and A: Tonsillectomy and adenoidectomy

## Testing and Diagnosis

### Computed tomography

Demonstrate the upper airway of a patient with OSA is different than the upper airway of a normal individual and might contribute to the diagnosis of OSA.[27]

### Pulse Oximetry

Screening for OSA using pulse oximetry has yielded conflicting results; in some reports, sensitivity and specificity is low while others report screening oximetry may be useful.[28,29]

### Polysomnography

The diagnosis of OSA requires a sleep study known as polysomnography. Polysomnography qualifies the number and duration of respiratory events occurring during sleep (apnoea, hypopnea).[30] An individual sleeps under observation and is specially monitored. Monitors include the following: ECG (ischaemia, arrhythmias), EEG (to assess and quantify different sleep stages), EMG (to correlate changes in muscle tone with EEG pattern), chest movement (using plethysmography to quantify respiratory effort), oronasal airflow, and pulse oximetry. Chest wall motion in combination with oronasal airflow can be used to distinguish between central and obstructive apnoea; the presence of chest motion during cessation of airflow at the nose is consistent with airflow obstruction. Apnoea is defined as no airflow for >10 seconds and hypopnea is defined as a reduction in airflow by greater than 50 percent for > 10 seconds. The total number of apnoea and hypopnea episodes per hour are quantified as per the apnoea hypopnea index (AHI) or the respiratory disturbance index (RDI). Also quantified are the number of episodes of desaturation, the degree of desaturations, and arrhythmias, if any. Mild sleep apnoea is defined as an AHI between 5 and 15, whereas severe OSA occurs when the AHI exceeds 30.[30] Doing polysomnography with CPAP may identify the patients who are likely to benefit from postoperative CPAP therapy.

### Associated diseases

There is an association between the presence of OSA and the following diseases:

- Hypertension[31,32]
- Obesity
- Coronary artery disease, myocardial dysfunction, and arrhythmias[33–35]
- Pulmonary arterial hypertension[36]
- Gastroesophageal reflux[37,38]

## Grading / Severity / Perioperative risk[39]

**Figure 2:**
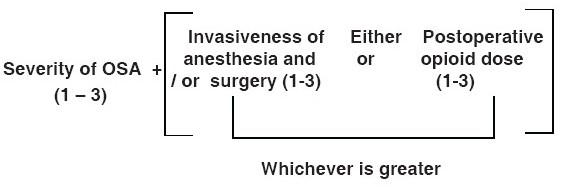
Calculating perioperative risk

The following scoring system may be used as a guide to estimate whether a patient is at increased perioperative risk of complications from obstructive sleep apnoea. However, clinical judgment should be used to assess the risk of an individual patient.

(A) Severity of sleep apnoea based on sleep study (i.e., apnoea- hypopnea index) or clinical indicators if sleep study is not available (i.e., presumptive diagnosis): none = 0; 1 = mild OSA; 2 = moderate OSA; 3 = severe OSA. One point may be subtracted if a patient has been on CPAP or bi-level positive airway pressure (BiPAP) prior to surgery and will be using his / her appliance consistently during the postoperative period. One point should be added if a patient with mild or moderate OSA also has a resulting PaCO_2_ > 50 mm Hg.

(B) Invasiveness of surgical procedure and anesthesia. Type of surgery / anesthesia: 0 = superficial surgery under local or peripheral nerve block anesthesia without sedation; 1 = superficial surgery with moderate sedation or general anesthesia or peripheral surgery with spinal or epidural anesthesia (with no more than moderate sedation); 2 = peripheral surgery with general anesthesia or airway surgery with moderate sedation; 3 = major surgery under general anesthesia or airway surgery under general anesthesia.

(C) Requirement for postoperative opioids: 0 = none; 1 = low - dose oral opioids; 2 = moderate dose oral opioid; 3 = high-dose oral opioids or parenteral or neuraxial opioids.

(D) *Estimation of perioperative risk is based on the overall score* = A + the degree of B or C points (0-6). Patients with overall score of 4 or greater may be at significantly increased perioperative risk from OSA [Figure 2].

*[*Patients *who are at significantly increased risk of perioperative complications (score ≥ 5) are generally not good candidates for ambulatory surgery. It is well accepted that patients with mild OSA undergoing superficial or minor surgical procedures under local, regional or general anesthesia as well as expected to have minimal postoperative opioid requirement may undergo ambulatory surgery.]*

## Preoperative Assessment and Preparation

Although polysomngraphy remains the gold standard in the diagnosis of OSA, it may not be always available. Therefore, a presumptive clinical diagnosis can be derived from:

(A) *History* of sleep disordered breathing (SDB), arousal from sleep, daytime somnolence, airway difficulty with previous anaesthetics, and morning headaches.

(B) *Physical* examination including body mass index, neck circumference (>17 inches in males and >16 inches in females), and presence of co-morbidities. Furthermore, it should also include an evaluation of the airway, nasopharyngeal characteristics, as well as tonsil and tongue volume.

The importance of presumptive clinical diagnosis of OSA lies with (a) preprocedure identification,[40] (b) inspire the caregivers to delay surgery, confirm and quantitate the diagnosis with a sleep study,[40] (c) to prepare an appropriate perioperative management plan.[40]

Perioperative optimization with CPAP or BiPAP therapy should be considered, particularly if OSA is severe. Patients who use CPAP devices at home should be advised to bring their devices to the facility for postoperative use. In addition to CPAP, other treatment modalities such as corrective surgery, mandibular advancement devices and oral appliances should be considered when feasible. It is recommended that a patient who had corrective airway surgery should be assumed to remain at risk for OSA complications unless the sleep studies and symptoms have normalized.[40]

The patients with OSA may be exquisitely sensitive to all central nervous system depressant drugs, with a potential for upper airway obstruction or apnoea with even minimal doses of these drugs. For these reasons, preoperative medication with sedatives including benzodiazepines or opioids should be used sparingly if at all.[41,42]

## Intraoperative Management

Intraoperative concerns in patients at increased perioperative risk from OSA include (1) choice of anaesthetic technique, (2) airway management, and (3) patient monitoring.

Local or regional anesthesia should be preferred whenever possible. Regional anesthesia does minimize the effect of anaesthetic agents on respiratory drive, can potentially reduce or eliminate the impact of anesthetic agents on subsequent sleep apnoeas, as well as maintain arousal responses during apnoeic episodes, and can potentially avoid airway management issues. For patients requiring moderate sedation, ventilation should be continuously monitored using capnography because of the increased risk of undetected airway obstruction in these patients. In patients using CPAP preoperatively, use of CPAP during moderate sedation may be beneficial. General anesthesia with a secure airway is preferable to deep sedation without a secure airway, particularly for procedures that may mechanically compromise the airway. Commonly used anaesthetic drugs such as thiopentone, propofol, opioids, benzodiazepines, and nitrous oxide all may reduce the tone of the pharyngeal musculature that acts to maintain airway patency.[13,43] Children with OSA and tonsillar hypertrophy have a diminished response to hypercarbia during halothane anesthesia and opioid may precipitate apnoea in intubated spontaneously breathing children.[44,45]

Not all patients with OSA are difficult to intubate. However, patients with OSA can be assumed to have redundant or excess oropharyngeal tissue, macroglassia, and so on. Thus, considerable care should be exercised in the approach to the airway management in these patients. If an “awake” tracheal intubation is planned, sedatives and opioids must be utilized judiciously. Thus, proper preparation should depend on thorough topical and nerve block anesthesia of the upper airway. If intubation is to be done with the patient asleep, it is important to fully preoxygenate the patient because the obese patient with a relatively small functional residual volume and high oxygen consumption desaturates much more rapidly during obstructive apnoea compared to a normal patient.[46,47] Laryngoscopy must be performed in the optimal “sniff” position. Mask ventilation must be performed optimally, which may require two anesthesia providers using two or three-handed bilateral jaw thrust and mask seal. Options to come out of “cannot ventilate, cannot intubate” situations must be immediately available at the anaesthetizing location.

### Extubation (awake versus leaving tube in situ)

The risk of airway obstruction following extubation is increased in OSA patients.[48] The risk is further increased in OSA, patients who have had nasal packing following nasal surgery,[49] therefore packing around a nasopharyngeal airway (creating a central conduit for gas exchange) should be considered.[50] Aside from the threat of death from airway obstruction, another great danger of spontaneous ventilation against an obstructed airway is rapid development of severe negative pressure pulmonary oedema.[51]

Because opioids may be associated with pronounced respiratory depression, patients with OSA benefit from prophylactic multimodal analgesia technique using nonopioid analgesics including local / regional anesthesia, acetaminophen, NSAIDs, ketamine and alpha-2- agonists. Residual muscle relaxation should be adequately antagonized, otherwise, minimal muscle relaxant can affect the airway muscles and result in airway obstruction. Unless there is a medical or surgical contraindication, patients at increased perioperative risk from OSA should be extubated while awake[1,40] in a semi-upright position when possible.

Anaesthetic management should also take into account the possibility of coronary artery disease and systemic and pulmonary artery hypertension. Patients with sleep apnoea may have down regulation of alpha and beta-receptors and may not respond appropriately or as expected to vasoactive substances. Patients with OSA should not be left unattended once sedated.

### Monitoring (haemodynamic and invasive)

Monitoring modalities are determined by known medical conditions. Because patient with sleep apnoea may be at increased risk for coronary artery disease or myocardial dysfunction, monitoring for myocardial ischaemia and rhythm disturbances is appropriate.

Transesophageal echocardiography is increasingly being used for noncardiac surgery and may be useful in selected patients with sleep apnoea because it can provide insight into heart function and pulmonary artery pressures. If the patient with OSA has morbid obesity an intraarterial catheter may be helpful as noninvasive blood pressure monitoring is unreliable or not possible for technical reasons.

## Postoperative Pain Management

In the postoperative setting, sleep architecture is disturbed. During the first three days after surgery, pain scores are highest and deep stage 3 and 4 NREM and REM sleep are often suppressed. High levels of pain results in increased analgesic requirements thus, the danger of life-threatening apnoea during drug-induced sleep is increased. Opioids can cause airway obstruction by pharyngeal collapse, as well as a poor ventilatory response to the ensuing hypoxaemia and hypercapnia. Also deep stage 3 and 4 NREM and REM sleep are often suppressed during the first three postoperative days. In the next three days, deep REM sleep rebounds. During this phase of recovery the danger of life-threatening natural deep sleep-induced apnoea is increased. Thus, for separate in-series reasons (increased analgesic requirement followed by increased amount of REM sleep), the risk of prolonged apnoea during sleep is increased for approximately one week for the postoperative OSA patient.[52]

Regional analgesic techniques should be considered to reduce or eliminate the requirement for systemic opioids in patients with OSA. If neuraxial analgesia is planned, benefits should be weighed (improved analgesia, decreased need for systemic opioids) against risks (respiratory depression from rostral spread) of using an opioid or opioid – local anaesthetic mixture as compared with a local anaesthetic alone. If patient-controlled systemic opioids are used, continuous background infusions should be used with extreme caution or avoided entirely. Nonsteroidal anti-inflammatory drugs and other modalities (e.g. ice, transcutaneous electrical nerve stimulation) should be considered if appropriate to reduce opioid requirements. Concurrent administration of sedative agents (benzodiazepines, barbiturates) increases the risk of respiratory depression and airway obstruction.

Use of nasal CPAP in OSA patients may allow the use of systemic analgesic agents and reduce-haemodynamic changes, however, additional randomized clinical trials are required to further define the role of CPAP in this setting.

## Postoperative Care

Although supplemental oxygen may be beneficial for most patients, it should be administered with caution as it may reduce hypoxic respiratory drive and increase the incidence and duration of apnoeic episodes. Recurrent hypoxaemia may be better treated with CPAP along with oxygen rather than oxygen alone.[1] It is recommended that patients who use CPAP preoperatively should use CPAP postoperatively, as it may reduce the risk of airway obstruction and respiratory depression.[40,51] Actually, even in absence of sleep apnoea, CPAP improved oxygenation after abdominal surgery.[53] Continuous positive airway pressure, however, should be used only after patients are awake, alert, and feasible (e.g. when patients are not ambulating). It is necessary that anaesthesiologists familiarize themselves with the use of CPAP devices, as determination of optimal CPAP setting may be difficult in patients who have not previously used CPAP.

If possible, patients should be placed in non supine positions (head end of bed raised 30°) throughout the recovery process. Continuous monitoring should be maintained as long as patients remain at increased risk. Intermittent pulse oximetry or continuous bedside oximetry without continuous observation does not provide the same level of safety.[40] When all the monitored factors in table 2 are mild, then the patient may reasonably go to a relatively unmonitored environment and when the factors mentioned in Table 2 are severe, the patient should go to an ICU. The large grey zone in between these extremes require careful judgment in “observational” (electronically and visually) units with nurse to patient ratios of 2 to 3: 1 [Table 2].[54]

**Table 2:** Determinants of location (Ward Vs ICU) for extubating obese OSA patient

Individual factors	Status of factors	Location
* Severity of BMI and AHI	All factors mild	Ward
* Associated cardio-pulmonary disease	One factors severe	ICU
* Narcotic / Sedative	In between the above extremes	Judgement → Observation unit

The ASA “Practice Guidelines for the perioperative management of patients with obstructive sleep apnoea” suggest OSA patients be monitored for a three hours longer than their non-OSA counterparts before discharge from the facility. In addition, the monitoring should continue for a median of seven hours after the last episode of airway obstruction or hypoxaemia while breathing room air in an unstimulated environment.[40]

The risks of caring for these challenging patients in the ambulatory venue are further amplified by the unfortunate fact that 80 to 90 percent of patients with OSA are undiagnosed,[40] they have neither a presumptive clinical and / or a sleep study diagnosis of OSA. This is concern because these patients may suffer perioperatively from life-threatening desaturation and postoperative airway obstruction. Moreover, presence of serious comorbidities like dysrhythmias, increased pulmonary artery pressure, corpulmonale, and congestive heart failure make these patients more critical from anaesthetic point of view.

## Conclusion

There is uncertainty regarding perioperative management of OSA patients. With limited understanding of their postoperative course, any recommendations remain speculative. However, conclusion has been drawn based upon few definitive data that exist to guide perioperative management of patients with OSA:

- Have high index of suspicion with obesity
- Identify and quantify comorbid disease(s)
- Perform meticulous airway assessment
- Have low threshold for awake intubation
- Administer sedative – hypnotics and narcotics sparingly
- Use short acting anaesthetic drugs
- Administer multimodal analgesics
- Extubate only when patient is fully awake
- Be able to administer CPAP.
